# The environmental correlates of overall and neighborhood based recreational walking (a cross-sectional analysis of the RECORD Study)

**DOI:** 10.1186/1479-5868-11-20

**Published:** 2014-02-21

**Authors:** Basile Chaix, Chantal Simon, Hélène Charreire, Frédérique Thomas, Yan Kestens, Noëlla Karusisi, Julie Vallée, Jean-Michel Oppert, Christiane Weber, Bruno Pannier

**Affiliations:** 1 Inserm, U707, 27 rue Chaligny, 75012 Paris, France; 2 Université Pierre et Marie Curie-Paris6, UMR-S 707, 27 rue Chaligny, 75012 Paris, France; 3 Lyon University, Inserm U870, Inra U1235, CRNH Rhône-Alpes, Chemin du Grand Revoyet, 69310 Pierre Bénite, France; 4 Université Paris-Est, Lab’Urba - Institut d’Urbanisme de Paris, 61 Avenue du général de Gaulle, 94010 Créteil, France; 5 Centre d’Investigations Préventives et Cliniques, 6 rue La Pérouse, 75116 Paris, France; 6 Social and Preventive Medicine Department, Université de Montréal, 7101 avenue du Parc, Montreal, QC H3N 1X7, Canada; 7 CNRS, UMR Géographie-cités, 13 rue du Four, 75006 Paris, France; 8 Inserm U557, Inra U1125, CNAM, EA3200, University Paris13, 74 rue Marcel Cachin, 93000 Bobigny, France; 9 Department of Nutrition, Pitie-Salpetriere Hospital (AP-HP), CRNH IdF, University Pierre et Marie Curie-Paris6, 47-83, boulevard de l’Hôpital, 75013 Paris, France; 10 ERL7230 CNRS Image, Ville, Environnement, 3 rue de l’Argonne, 67000 Strasbourg University, Strasbourg, France

**Keywords:** Walking, Recreational activity, Neighborhood environment, Physical and social contexts, Spatial analysis, Geographic Information Systems

## Abstract

**Background:**

Preliminary evidence suggests that recreational walking has different environmental determinants than utilitarian walking. However, previous studies are limited in their assessment of environmental exposures and recreational walking and in the applied modeling strategies. Accounting for individual sociodemographic profiles and weather over the walking assessment period, the study examined whether numerous street network-based neighborhood characteristics related to the sociodemographic, physical, service, social-interactional, and symbolic environments were associated with overall recreational walking and recreational walking in one’s residential neighborhood and could explain their spatial distribution.

**Methods:**

Based on the RECORD Cohort Study (Paris region, France, n = 7105, 2007–2008 data), multilevel-spatial regression analyses were conducted to investigate environmental factors associated with recreational walking (evaluated by questionnaire at baseline). A risk score approach was applied to quantify the overall disparities in recreational walking that were predicted by the environmental determinants.

**Results:**

Sixty-nine percent of the participants reported recreational walking over the past 7 days. Their mean reported recreational walking time was 3h31mn. After individual-level adjustment, a higher neighborhood education, a higher density of destinations, green and open spaces of quality, and the absence of exposure to air traffic were associated with higher odds of recreational walking and/or a higher recreational walking time in one’s residential neighborhood. As the overall disparities that were predicted by these environmental factors, the odds of reporting recreational walking and the odds of a higher recreational walking time in one’s neighborhood were, respectively, 1.59 [95% confidence interval (CI): 1.56, 1.62] times and 1.81 (95% CI: 1.73, 1.87) times higher in the most vs. the least supportive environments (based on the quartiles).

**Conclusions:**

Providing green/open spaces of quality, building communities with services accessible from the residence, and addressing environmental nuisances such as those related to air traffic may foster recreational walking in one’s environment.

## Background

In physical activity promotion, walking is receiving increasing attention [1-5]. Studies suggest that utilitarian and recreational walking have distinct environmental determinants [6-11]. The most commonly examined environmental determinants of walking pertain to the structural walkability of neighborhoods (related to land use mix, street connectivity, and density), i.e., the extent to which the local street network is walkable and allows one to access to local destinations [6,10-21]. Factors related to the pleasantness/unpleasantness of the environment that may be important for recreational walking have received less attention, were often assessed as individual perceptions [22,23] rather than at the neighborhood level [24,25], and were restricted to a limited number of dimensions (e.g., safety and esthetics). To address this gap, the present study on recreational walking investigates a wide set of environmental factors that include, in addition to the usual walkability dimension, factors related to neighborhood esthetic and pleasantness (e.g., the absence of highways, traffic-related pollution, waste treatment facilities, stressful social interactions and the presence of monuments or social cohesion; see Table 1). These environmental factors, selected on the basis of definite hypotheses (Table 2), pertain to the sociodemographic, physical, service, social-interactional, and symbolic environments [26,27] (see Table 1). It is relevant to focus on recreational walking, as environmental interventions to promote utilitarian walking may not be particularly efficient for recreational walking.

**Table 1:** Characteristics of sociodemographic, physical, service, social-interactional, and symbolic environments as possible correlates of recreational walking

**Neighborhood characteristic**	**Data**	**Measurement approach**
**Neighborhood sociodemographic environment**		
Neighborhood median income	Exhaustive data from the Tax Registry of DGI in 2006 geocoded at the residential address by Insee	Aggregation of population data within street network buffers^a^: median household income per consumption unit
Neighborhood education	Population Census of 2006 geocoded at the residential address by Insee	Aggregation of population data within street network buffers^a^: proportion of residents with University education
Neighborhood population density	Population Census of 2006 geocoded at the residential address by Insee	Aggregation of population data within street network buffers^a^: number of inhabitants per km^2^
**Neighborhood physical environment**		
Proportion of the neighborhood covered with buildings	3-dimensional data from IGN on buildings’ ground shape and height in 2008	GIS processing: proportion of built surface within street network buffers^a^
Surface of green spaces	Linear and polygonal data from IAU-IdF on public parks and green spaces in 2008	GIS processing: proportion of surface covered with green spaces within street network buffers^a^
Presence of a lake or waterway	Polygonal data from IAU-IdF on land use in 2003	GIS processing: presence of water in street network buffers^a^
Density of street intersections	Data on the street network in 2008 from IGN	GIS processing: count of intersections with at least 3 ways within street network buffers^a^
Link node ratio	Data on the street network in 2008 from IGN	GIS processing: number of links divided by the number of nodes within street network buffers^a^
Highway nearby the dwelling	Data on the street network in 2008 from IGN	GIS processing: presence of a highway within 250 m (straight-line distance)
Road traffic-related pollution (nitrogen dioxide)	Modeled data from AIRPARIF on annual concentrations of nitrogen dioxide in 2007-2008	GIS processing: average concentration within street network buffers^a^
Air traffic exposure area	Data on air traffic from ACNUSA in 2005	GIS processing: air traffic below 2000 m in the street network buffers
Waste treatment facilities	Geocoded waste treatment facilities in 2008 from IAU-IdF	GIS processing: presence of a waste treatment facility in street network buffers^a^
Presence and quality of green and open spaces	3 items from the RECORD questionnaire	3-level multilevel ordinal ecometric model (TRIRIS neighborhood)
Deterioration of the physical environment	4 items from the RECORD questionnaire	3-level multilevel ordinal ecometric model (TRIRIS neighborhood)
**Neighborhood service environment**		
Density of destinations	Geocoded destinations from the 2008 Permanent Database of Facilities of Insee	GIS processing: count of destinations (administrations, public/private shops, entertainment facilities, etc.) within street network buffers^a^
Presence of monuments	Geocoded monuments in 2005 from IAU-IdF	GIS processing: count of monuments within street network buffers^a^
Number of transportation lines	Geocoded stops of buses, metros, and trains in 2008 from STIF	GIS processing: count of different lines within street network buffers^a^
Proportion of incoming and outgoing traffic by public transportation rather than car	Outputs of a road traffic model from DRE-IdF	GIS processing: proportion of traffic by public transportation in the residential area
Presence of a shopping center	Geocoded shopping centers in 2008 from IAU-IdF	GIS processing: presence of a shopping center within street network buffers^a^
**Neighborhood social interactions**		
School violence nearby the dwelling	School violence in 2005-2006 from the Ministry of Education	Multilevel modeling of violence behavior in schools and GIS processing: average violence in schools nearby home
Neighborhood social cohesion	4 items from the RECORD questionnaire	3-level multilevel ordinal ecometric model (TRIRIS neighborhood)
Neighborhood shared feeling of e insecurity	1 item from the RECORD questionnaire	2-level multilevel ordinal ecometric model (TRIRIS neighborhood)
Neighborhood stressful social interactions	5 items from the RECORD questionnaire	3-level multilevel ordinal ecometric model (TRIRIS neighborhood)
Neighborhood mistrust and hostility	5 items from the RECORD questionnaire	3-level multilevel ordinal ecometric model (TRIRIS neighborhood)
**Neighborhood symbolic environment**^b^		
Stigmatized neighborhood identity	3 items from the RECORD questionnaire	3-level multilevel ordinal ecometric model (TRIRIS neighborhood)

Legend: ACNUSA, Authority for the Control of Airport Nuisances; DGI, General Directorate of Taxation; DRE-IdF: Direction of Equipment and Infrastructures of Ile-de-France region; IAU-IdF, Institute of Urban Planning of the Ile-de-France region; IGN, National Geographic Institute; Insee, National Institute of Statistics and Economic Studies; GIS, Geographic Information System; STIF, the Ile-de-France Transportation Authority.

^a^Variables within street network buffers were determined with a radius of 1000 m.

^b^The symbolic environment refers to the territorial identities associated with each neighborhood.

**Table 2:** A priori hypotheses of effects for the environmental variables examined

**Variable**	**Expected direction**	**Hypothesis**
**Neighborhood sociodemographic environment**		
Neighborhood median income	Positive	Nicer, cleaner, and safer environments in affluent neighborhoods promote recreational walking
Neighborhood education	Positive	A high average education in the neighborhood may stimulate values that are favorable to a healthy and physically active lifestyle
Neighborhood population density	Positive	A high population density was hypothesized to encourage walking according to the walkability hypothesis (e.g., easiness of walking to visit members of one’s social network)
**Neighborhood physical environment**		
Proportion of the neighborhood covered with buildings	Positive	A high density of buildings promotes walking through shorter distances to destinations
Surface of green spaces	Positive	Green spaces provide a pleasant context for recreational walking
Presence of a lake or waterway	Positive	Lakes/waterways are an enjoyable environmental feature when walking
Density of street intersections	Positive	Denser street networks and related shorter distances are more walkable
Link node ratio	Positive	More connected street networks represent more walkable neighborhoods
Highway nearby the dwelling	Negative	Due to noise and smell, a highway is unpleasant for recreational walking
Road traffic-related pollution (nitrogen dioxide)	Negative	Road traffic is a source of noise and unpleasant smells and is potentially dangerous.
Air traffic exposure area	Negative	Air traffic noise is a source of annoyance when walking
Waste treatment facilities	Negative	Waste treatment facilities may be associated with unpleasant smells as a source of annoyance
Presence and quality of green and open spaces	Positive	Green and open spaces of quality provide a pleasant context for recreational walking
Deterioration of the physical environment	Negative	A deteriorated physical environment may discourage recreational walking
**Neighborhood service environment**		
Density of destinations	Positive	A high density of services promotes walking, even when people have no definite purchase intentions as in recreational walking
Presence of monuments	Positive	Monuments are enjoyable environmental features that foster recreational walking
Number of transportation lines	Positive	A high number of transportation lines facilitates access to enjoyable places for walking. A high number of transportation lines may also be a marker of an attractive neighborhood
Proportion of incoming and outgoing traffic by public transportation rather than car	Positive	Places with a higher share of trips by public transport represent more walkable neighborhoods
Presence of a shopping center	Positive	Shopping centers are a common destination for recreational walking
**Neighborhood social interactions**		
School violence nearby the dwelling	Negative	Fear of violence and crime discourages from walking
Neighborhood social cohesion	Positive	Socially cohesive neighborhoods provide a pleasant context for walking
Neighborhood shared feeling of insecurity	Negative	Fear of violence and crime discourages from walking
Neighborhood stressful social interactions	Negative	Fear of incivilities discourages from walking
Neighborhood mistrust and hostility	Negative	Mistrust and hostility among neighbors discourage from walking
**Neighborhood symbolic environment**		
Stigmatized neighborhood identity	Negative	Neighborhoods with a stigmatized identity are not attractive for walking

**Figure 1:**
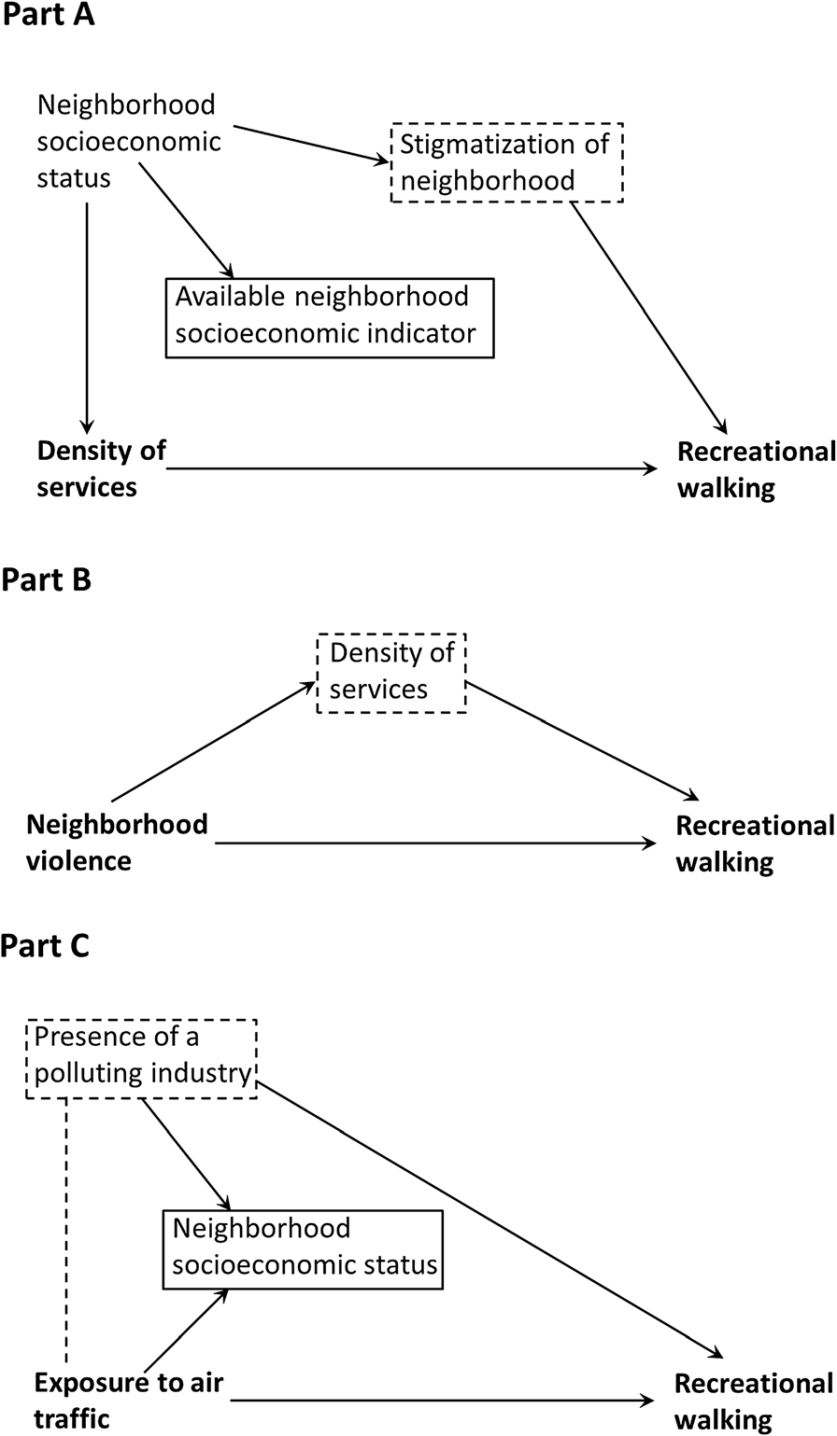
**Directed Acyclic Graphs depicting confounding (Part A), an indirect biasing pathway (Part B), and a collider-stratification bias (Part C) in the estimation of relationships between environmental factors and recreational walking.** The relationship of interest is represented in bold. A solid arrow indicates a causal effect. A dotted line indicates a spurious association generated through adjustment. A solid box around a factor indicates that it is adjusted for. A dotted box around a factor indicates that it should be adjusted for.

Although considering a large number of environmental factors increases the risk of spurious associations, it is critical to examine concurrently the different factors, both to reach a comprehensive appraisal of the multifactorial environmental influences on walking and to disentangle the effects of the different factors. Investigating few environmental determinants in isolation of the others may also lead to spurious or incorrectly adjusted associations [28], as shown in the Directed Acyclic Graphs (DAG) of hypothetical scenarios inspired from the study reported in Figure 1. The first DAG (Part A) shows that neighborhood socioeconomic status generates confounding for the relationship between the density of destinations and walking through a backdoor path involving neighborhood stigmatization [28]. The DAG illustrates that adjusting for neighborhood socioeconomic status is not sufficient [29], because our neighborhood socioeconomic indicators may imperfectly capture the exact socioeconomic status components influencing the density of destinations and stigmatization. It is therefore important to adjust for the various causal descendants of socioeconomic status that influence walking.

Part B in Figure 1 shows that it is also important to adjust for “indirect biasing pathways” [28]. In this example, the density of services mediates part of the effects of violence on walking. However, the path from violence through the density of services may be seen as an “indirect biasing pathway”, because the violence effect we are interested to isolate operates through fear of walking in one’s neighborhood (i.e., net of the density of services effects on walking).

Finally, adjusting for a given environmental factor may generate rather than remove bias [28]. In Figure 1 (Part C), both air traffic exposure and the presence of a polluting industry influence neighborhood socioeconomic status through the emigration/immigration of affluent/poor people. If the presence of polluting industries is not assessed in the study, conditioning on neighborhood socioeconomic status generates a spurious relationship between air traffic exposure and the presence of a polluting industry that biases the estimated air traffic exposure effect on walking (collider-stratification bias). In the present study, as it was critical to control for neighborhood socioeconomic status (Figure 1, Part A), it was important to identify environmental variables that could generate collider-stratification biases if not adjusted for as in Figure 1 (Part C). Overall, these three DAGs suggest that it is relevant to correctly adjust models for other environmental factors to identify unbiased and specific environmental effects on walking.

The study also attempted to assess the overall magnitude of disparities in walking that were predicted by the different environmental factors. Quantifying such disparities is useful to inform policy makers on whether physical activity promotion efforts should focus or not on environmental interventions.

Finally, the study was able to investigate the probability of walking and location of recreational walking [30,31] as recently recommended [11] and applied spatial-multilevel regression [32,33]. Identification of between-neighborhood variations with classical multilevel models is an indication that some processes that vary from one neighborhood to the other may influence walking [34]. Assessment of spatial autocorrelation in walking, i.e., of whether neighborhoods located nearby have more similar recreational walking habits that neighborhoods located further apart, is an additional step in the investigation of the geographic variability. It allows one to identify coherent patterns of variability in walking over space, which is useful to generate hypotheses on the underlying environmental predictors, based on the fact that many environmental factors are themselves strongly autocorrelated. The spatial-multilevel regression models that were estimated, which have never been used in physical activity research, allowed us to assess both within-neighborhood correlation and spatial autocorrelation in walking. Using this modeling approach, our aim was to test the hypotheses of environmental effects on recreational walking reported in Table 2 and assess the extent to which the individual/environmental determinants investigated explained the spatial distribution of recreational walking.

## Methods

### Population

The RECORD Cohort Study [35,36] includes 7290 participants recruited in 2007–2008 during 2-hour-long preventive medical checkups conducted by the Centre d’Investigations Préventives et Cliniques in 4 of its health centers in the Paris metropolitan area [37-42]. These checkups are offered for free to all working and retired employees and their families. We recruited without *a priori* sampling people who were attending the healthcare centers without invitation from our part (convenience sample). The eligible population for these preventive health checkups includes all the currently working, unemployed, and retired salaried workers and their families. In our study counties, this group represents 95% of the overall population [36]. However, the recruitment channels of these healthcare centers are very diverse (people’s own initiative or appointments through the employers, work physicians, social workers, various associations, etc.). The absence of randomization in the recruitment of the participants led to a sample that was not representative of the background population. A previous work showed that a high individual education, a high neighborhood socioeconomic status, and a low building density were associated with higher odds of participation in the RECORD Study [38]. All these factors were included in the models or considered for adjustment to minimize bias.

Eligibility criteria were as follows: age 30 to 79 years, ability to fill out questionnaires, and residence in one of the 10 (out of 20) administrative divisions of Paris or 111 other municipalities of the Paris Ile-de-France region (among a large number of municipalities in the region) that were selected *a priori*. The districts and municipalities were selected among those that provided a large number of consultants to the medical center in the years prior to the recruitment, and in an attempt to maximize municipality-level socioeconomic disparities and to cover both urban and periurban territories. Of the eligible participants, 83.6% accepted to participate and completed the data collection protocol.

Participants were geocoded based on their residential address in 2007–2008, using the geocoding tool of the French National Institute of Statistics and Economic Studies that ensured an exact correspondence between the spatial coordinates and census tract neighborhoods. Research assistants corrected all incorrect or incomplete addresses with the participants by telephone, and extensive investigations with local departments of urban planning were conducted to complete the geocoding when needed. The study protocol was approved by the French Data Protection Authority. After excluding individuals with missing values for walking (n = 185, see Additional file 1A), 7105 participants from 661 census tracts (TRIRIS areas) were included in the analyses.

### Measures

#### Recreational walking

The questionnaire to collect walking data, developed by ourselves, relied on a 7-day recall period, as in the question on walking of the Short form of the International Physical Activity Questionnaire (IPAQ-SF) [43]. In our baseline questionnaire, participants were asked to report retrospectively the number of hours and minutes they had walked over the previous 7 days, separately for home-work commuting, shopping, going to other destinations, and leisure. Listing different types of destinations or purposes of walking served as a prompt to facilitate the recall of walking episodes. For each of the walking categories, participants had to distinguish between walking time within and outside their residential neighborhood, assessed according to each participant’s subjective perception of her/his self-defined neighborhood (neither participants were provided objective indications on the size of the neighborhood to consider [31], nor were they asked to objectify how they perceived it). Our expectation was that this instrument, even if imprecise, should be able to discriminate between participants who make most of their recreational walking in their neighborhood and participants who make most of their recreational walking far from their neighborhood.

Two complementary outcomes were defined: (i) reporting any recreational walking or not (coded as a binary variable), in order to assess the overall practice of recreational walking; and (ii) the reported recreational walking time made in one’s residential neighborhood (coded as a 5-category ordinal variable with the first category corresponding to value 0 and the other 4 categories comprising a similar number of participants).

#### Individual variables

Age was divided in 3 classes (30-44, 45-59, and 60-79 years). Gender and cohabitation status (living alone or as a couple) were coded in two classes. Education was divided in four classes: no education; primary education and lower secondary education; higher secondary education and lower tertiary education; and upper tertiary education. Employment status was coded in three categories: employed, unemployed, and retired. Four occupational categories were distinguished: blue collar workers; low white collar workers; intermediate occupations; and high white collar workers. Household income adjusted for household size was divided into four categories. Binary variables were determined for perceived financial precariousness, self-reported financial strain, and dwelling ownership. The 2006 Human Development Index of each participant’s country of birth [44] was used to distinguish participants born in France from those who were born in low, intermediate, and high development countries.

#### Weather variables

Daily meteorological data in the Ile-de-France region were provided by Meteo France for 2007–2008. Average weather variables were defined for each participant for the recruitment day and 7 previous days (the variables allowed us to compare participants recruited in different time periods over 1 year). Different aspects of meteorological condition were considered (in 4 categories based on the quartiles): minimum temperature; maximum temperature; average temperature; rainfall; wind speed; sunshine duration; presence of fog; and presence of mist.

#### Residential neighborhood measures

The 25 environmental factors investigated are summarized in Table 1. As noted in Table 1, most variables were determined, using a Geographic Information System (GIS), within street network-based neighborhoods centered on participants’ residences. Based on sensitivity analyses performed in the RECORD Study on the optimal street network distance for assessing the accessibility to facilities [45], these variables were determined within street network buffers of 1000 m of radius. This radius is also coherent with the idea that 1000 m along the street network represent a relatively easily walkable distance to services in urban environments. To ensure their replicability, these environmental variables were determined with Python scripts based on ArcInfo 10, with all procedures encoded in a text file.

Other contextual variables were determined with the ecometric approach [46,47]. They were defined at the level of TRIRIS neighborhoods that comprised a median number of 7980 inhabitants in 2006. Briefly, three-level (survey questions, individuals, TRIRIS areas) multilevel models were estimated with the RECORD participants’ answers to different questions about their neighborhood as the outcome. These multilevel models were used to aggregate the survey information at the neighborhood level, to derive neighborhood variables such as the presence/quality of green/open spaces or the degree of stressfulness of social interactions (see Additional file 1B for a description of the ecometric approach).

Most environmental variables were divided into 4 categories comprising a similar number of participants, to assess whether associations are monotonous, and if so linear (see Additional file 1C for the cutoffs).

### Statistical analysis

The present article reports cross-sectional analyses of baseline data of the RECORD Study. Reporting recreational walking or not was modeled with a logistic regression. The recreational walking time in the neighborhood was analyzed with a logit ordinal model. Coding the neighborhood recreational walking time as a continuous variable led to non-normal residuals in a linear model even after adjustment, while coding it as a binary variable would imply a substantial loss of information.

Multilevel-spatial regression models were estimated [32,48,49], with two distinct random effects at the census tract neighborhood level incorporated in the same model [32]: both a classical random effect to model unstructured between-neighborhood variations, and a conditional autoregressive Normal random effect to account for spatially structured patterns of variability, i.e., correlation in walking between adjacent neighborhoods. Unstructured and spatially structured neighborhood variations (i.e., two components of the between-neighborhood variance that reflect patterns of variations that respectively show and do not show spatial autocorrelation) were expressed with interquartile odds ratios [32,50,51]. Such ratios quantify on the odds ratio scale the difference in walking between the 25% of individuals in neighborhoods with the lowest prevalence of recreational walking and the 25% of individuals in neighborhoods with the highest prevalence of recreational walking. Assessing spatially structured patterns of variability in walking is useful to generate hypotheses on the underlying social and environmental processes at play [32,33,52]. We therefore derived maps of the spatially structured pattern of variations in walking at the different steps of the modeling, and calculated the proportion of the total between-neighborhood variance that was spatially structured [53] as an indication of whether such spatially autocorrelated pattern in walking accounted for a small part or substantial part of residual between-neighborhood variations.

First, models only adjusted for age and sex were estimated, to assess between-neighborhood variability in walking. Second, models retaining the individual and weather variables independently associated with each outcome were estimated. Third, on the basis of the previous models, all environmental variables were pretested one by one [39]. Fourth, the environmental variables independently associated with each outcome were progressively combined into one model for each outcome. Fifth, interactions were tested between the meteorological variables and the neighborhood variables retained in the models, in order to identify environmental conditions that are supportive of walking even under poor weather (which is relevant given the frequent poor weather conditions in our region). Models were estimated using Winbugs 1.4 [54].

To provide a sense of the overall disparities between individuals that were predicted by the environmental factors retained in the models, using the SAS software, we calculated a risk score of recreational walking as a function of the environmental predictors from the final models (sum of products of coefficients by variable values for each individual); we divided this risk score in four categories based on the quartiles; and we re-estimated the final models after replacing all the environmental variables by the risk score categories. For these categories, we report the median of the odds ratio and the 2.5^th^ and 97.5^th^ percentiles as confidence intervals over 2000 bootstrapped samples (analysis performed with SAS 9.3).

## Results

### Descriptive findings and initial regression models

The sociodemographic characteristics of the sample are reported in Table 3. Overall, 69% of the participants reported recreational walking. The mean recreational walking time among recreational walkers was 3h31mn (interquartile range: 1h00mn, 4h00mn). Seventy-four percent of the recreational walkers accomplished at least part of this walking activity in their residential neighborhood.

**Table 3:** Distribution of participants according to the main individual variables (n = 7105)

	**%**
*Age*	
30–44	35.5%
45–59	41.7%
60–79	22.9%
*Male*	65.6%
*Living alone rather than as a couple*	29.8%
*Individual education*	
No education	7.5%
Primary and lower secondary	24.1%
Higher secondary and lower tertiary	29.5%
Upper tertiary	38.1
*Employment status*	
Employed	61.7%
Unemployed	15.1%
Retired	17.7%
*Occupation*	
High white collar worker	39.7%
Intermediate occupation	5.5%
Low white collar worker	38.2%
Blue collar worker	11.0%
*Non-ownership of dwelling*	45.4%

**Figure 2:**
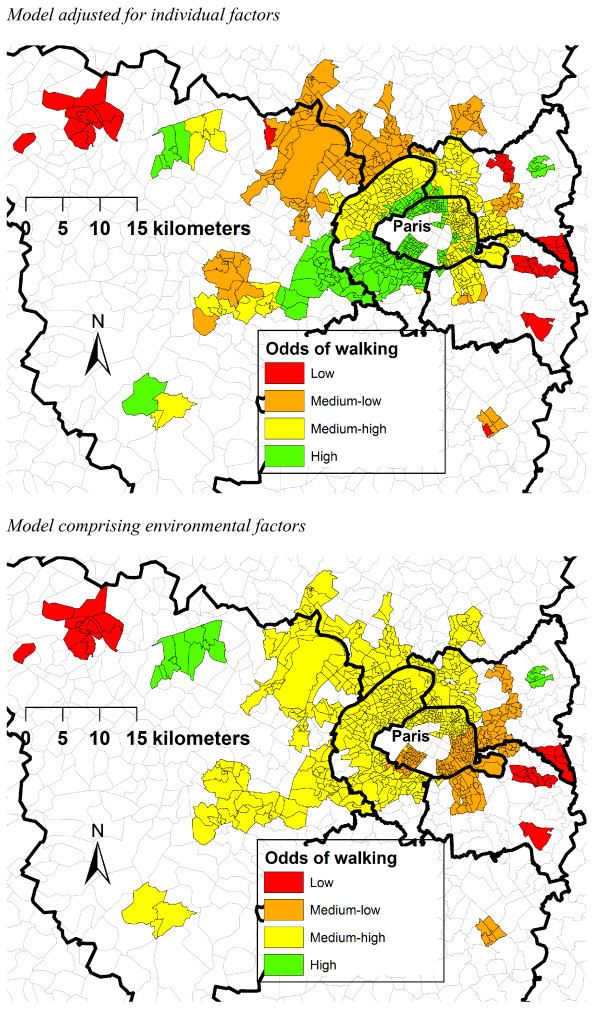
Spatially structured between-neighborhood variations in the odds of recreational walking, as assessed from spatial-multilevel regression models including individual and meteorological variables (Part A) and further including environmental factors (Part B).

After adjustment for age and gender, between-neighborhood variations were observed for the two walking outcomes (see Table 4). Between-neighborhood variations showed a relatively strong spatial structure for reporting or not recreational walking and for the recreational walking time in the neighborhood (i.e., over 60% of between-neighborhood variations were spatially structured, i.e., were attributable to a spatially autocorrelated rather than unstructured pattern of variability). The spatially structured component of between-neighborhood variations in the odds of reporting recreational walking is represented in Figure 2.

**Table 4:** **Spatially structured, spatially unstructured, and total between-neighborhood variations in recreational walking**^**a,b**^

	**IqOR for the spatially unstructured variance (95% CrI)**	**IqOR for the spatially structured variance (95% CrI)**	**IqOR for the total between-neighborhood variance (95% CrI)**	**Percentage of structured variance (95% CrI)**
**Recreational walking or not**				
Age and sex model	1.56 (1.35, 1.88)	1.77 (1.47, 2.13)	2.07 (1.76, 2.46)	62% (35%, 82%)
Individual-level model	1.58 (1.36, 1.92)	1.64 (1.36, 1.98)	1.97 (1.67, 2.36)	53% (25%, 78%)
Environmental model	1.57 (1.36, 1.88)	1.34 (1.20, 1.52)	1.71 (1.48, 2.03)	29% (12%, 54%)
**Recreational walking time in the neighborhood**				
Age and sex model	1.52 (1.33, 1.81)	1.85 (1.52, 2.23)	2.12 (1.83, 2.48)	68% (39%, 85%)
Individual-level model	1.53 (1.34, 1.82)	1.97 (1.67, 2.34)	2.24 (1.95, 2.61)	72% (49%, 86%)
Environmental model	1.61 (1.39, 1.89)	1.36 (1.20, 1.65)	1.77 (1.53, 2.09)	30% (11%, 60%)

Legend: CrI, credible interval; IqOR, interquartile odds ratio.

^a^The first model only included age and sex. The second model further introduced individual sociodemographic variables and weather variables. The third model further included the environmental variables associated with each outcome (see Tables 4 and 5).

^b^Regarding recreational walking or not, the IqOR of 2.07 for the total between-neighborhood variance in the age and sex model indicates that the 25% of participants living in neighborhoods with the highest odds of recreational walking had 2.07 times larger odds of recreational walking than the 25% of participants residing in neighborhoods with the lowest odds of recreational walking. In the same model, 62% of the total between neighborhood variance was attributable to the spatially structured component of neighborhood variations. When progressively adding covariates to the models, spatially structured variations decreased to a large extent but spatially unstructured variations did not, resulting in a decreasing share of the total between-neighborhood variability that was spatially structured.

Individual sociodemographic and weather variables were then introduced into the models (Tables 5 and 6). Low white collar workers and blue collar workers had lower odds to report recreational walking over 7 days. Higher average temperature and lower rainfalls were associated with a higher prevalence of recreational walking and with a higher neighborhood recreational walking time.

**Table 5:** **Associations of individual, weather, and environmental variables with reporting any recreational walking**^**a**^

	**OR (95% CrI)**
**Individual variables**	
*Age (vs. 30–44)*	
45–59	1.01 (0.90, 1.14)
60–79	1.02 (0.81, 1.27)
*Male (vs. female)*	1.38 (1.23, 1.55)
*Individual education (vs. upper tertiary)*	
Higher secondary and lower tertiary	1.12 (0.98, 1.29)
Primary and lower secondary	1.01 (0.86, 1.18)
No education	0.81 (0.65, 1.02)
*Employment status (vs. employed)*	
Unemployed	1.15 (0.99, 1.35)
Retired	1.68 (1.32, 2.15)
*Occupation (vs. high white collar)*	
Intermediate	0.83 (0.65, 1.06)
Low white collar	0.83 (0.72, 0.96)
Blue collar	0.79 (0.64, 0.97)
**Weather variables**	
*Rainfall over 7 days (vs. low)*	
Medium low	0.85 (0.73, 0.99)
Medium high	0.82 (0.71, 0.96)
High	0.78 (0.67, 0.90)
*Mean temperature over 7 days (vs. low)*	
Medium low	1.14 (0.99, 1.32)
Medium high	1.40 (1.20, 1.62)
High	1.37 (1.18, 1.59)
**Environmental variables**	
*Neighborhood education level (vs. low)*	
Medium low	1.17 (1.00, 1.36)
Medium high	1.45 (1.23, 1.71)
High	1.47 (1.24, 1.75)
*Air traffic exposure area*	0.75 (0.65, 0.87)

Legend: CrI, credible interval; OR, odds ratio.

^a^At each step of the modeling, only the additional variables tested that were independently associated with the outcome were retained in the model.

**Table 6:** **Associations of individual, weather, and environmental variables with recreational walking time in the residential neighborhood**^**a**^

	**OR (95% CrI)**
**Individual variables**	
*Age (vs. 30–44)*	
45–59	1.00 (0.90, 1.11)
60–79	0.95 (0.78, 1.14)
*Male (vs. female)*	1.28 (1.17, 1.42)
*Living alone (vs. as a couple)*	0.80 (0.72, 0.89)
*Employment status (vs. employed)*	
Unemployed	1.37 (1.20, 1.55)
Retired	1.89 (1.54, 2.31)
*Non-ownership of dwelling (vs. ownership)*	1.18 (1.07, 1.30)
**Weather variables**	
*Rainfall over 7 days (vs. low)*	
Medium low	1.02 (0.89, 1.16)
Medium high	0.89 (0.79, 1.01)
High	0.85 (0.75, 0.96)
*Mean temperature over 7 days (vs. low)*	
Medium low	0.99 (0.87, 1.12)
Medium high	1.17 (1.04, 1.33)
High	1.17 (1.03, 1.33)
**Environmental variables**	
*Neighborhood education level (vs. low)*	
Medium low	1.03 (0.88, 1.20)
Medium high	1.26 (1.06, 1.50)
High	1.21 (1.00, 1.48)
*Presence and quality of green and open spaces (vs. low)*	
Medium low	0.97 (0.85, 1.11)
Medium high	1.13 (0.98, 1.31)
High	1.43 (1.21, 1.70)
*Air traffic exposure area*	0.82 (0.71, 0.95)
*Density of destinations (vs. low)*	
Medium low	1.09 (0.95, 1.25)
Medium high	1.14 (0.96, 1.35)
High	1.39 (1.13, 1.70)

Legend: CrI, credible interval; OR, odds ratio.

^a^At each step of the modeling, only the additional variables tested that were independently associated with the outcome were retained in the model.

### Associations with residential neighborhood variables

Regarding the first outcome (Table 5), the likelihood of reporting recreational walking was higher in neighborhoods with a medium-high or high level of education compared to low education neighborhoods. Moreover, residing in an air traffic exposure area was related to a decreased probability of recreational walking, after adjustment for all the factors listed in Table 5.

Regarding the second outcome (Table 6), a higher neighborhood education level, the presence of green/open spaces of quality, and a higher density of destinations nearby were independently associated with a higher neighborhood recreational walking time. In the opposite direction, exposure to air traffic was related to a reduced walking time in one’s residential neighborhood.

No interaction was documented between the environmental variables and the weather variables retained in the final models.

As shown in Table 4, accounting for the environmental factors led to a notable reduction in the spatially structured between-neighborhood variations in walking, especially for the neighborhood recreational walking time but also for reporting recreational walking or not (i.e., the interquartile odds ratios quantifying the spatially autocorrelated pattern of variability decreased when environmental factors were added to the models). On the opposite, the environmental factors did not explain spatially unstructured between-neighborhood variations in walking (the corresponding interquartile odds ratios did not decrease when including environmental factors into the models). Before adjustment for environmental factors (top part of Figure 2), higher odds of recreational walking were documented in Paris and in the first crown of counties around Paris, especially in the westernmost county of the first crown of counties which is the most urbanized after Paris. As shown in the bottom part of Figure 2, the residual spatially structured patterns of variations in recreational walking were substantially altered after accounting for the higher density of destinations in Paris and in the first crown of counties, for the more frequent exposure to air traffic in the western and northern counties of the second crown of counties, and for the more frequent presence of green/open spaces of quality in the western counties of the first and second crowns of counties.

When all the environmental variables in each model were replaced by a risk score, it was found that, compared to the environments with the lowest environmental supportiveness of walking, the odds of reporting recreational walking were 1.17 (95% CI: 1.13, 1.20) times, 1.55 (95% CI: 1.44, 1.62) times, and 1.59 (95% CI: 1.56, 1.62) times higher in the three categories of increasing environmental supportiveness. The odds of reporting a higher recreational walking time in one’s neighborhood were 1.20 (95% CI: 1.13, 1.27) times, 1.52 (95% CI: 1.44, 1.60) times, and 1.81 (95% CI: 1.73, 1.87) times higher in these three categories of increasing environmental supportiveness.

## Discussion

Our study showed that several contextual factors related to the socioeconomic environment, physical environment, and service environment were associated with recreational walking. Although these factors contributed to generate substantial disparities in recreational walking over the study territory, there were residual geographic variations in this walking behavior that remained unexplained.

### Strengths and limitations

Strengths of the study include the multilevel-spatial modeling investigation performed, the numerous individual, environmental, and meteorological determinants considered, the determination of environmental variables in street network buffers using Python scripts that ensure replicability, and the two complementary variables analyzed to capture overall walking and walking in one’s residential neighborhood. Study limitations include its cross-sectional design and the declarative nature of walking data (a shortcoming that we address with GPS and accelerometers in the RECORD GPS and MultiSensor Studies [55,56]). Concerns related to declarative walking data include the fact that the different population groups may have a distinct definition of recreational walking, a different recall rate of recreational walking episodes, a different perception of the social desirability of regular walking, and a different accuracy in walking times assessment. Overall, measurement error may either dilute the associations reported or systematically bias some of them, e.g., those between neighborhood education and walking. Another limitation is the lack of adjustment for neighborhood selection factors that likely confound the reported associations [10,57-59]. For example, a high motivation for recreational walking leading certain participants to choose to reside, when moving, in a neighborhood with a lot of green spaces would confound the relationship between the spatial accessibility to green spaces and recreational walking.

Moreover, participants had to rely on a subjective definition of their neighborhood (i.e., no instructions were provided to the participants) to report (i) the perceptions of their neighborhood (that were used to determine the neighborhood ecometric variables) and (i) the location of walking. Regarding the first aspect, it seems relevant to adhere to the participants’ definition of neighborhood to assess their psychological relationship with it. Regarding the second aspect, however, using a subjective definition of neighborhood for the assessment of walking may have biased the analyses on the recreational walking time made in one’s residential neighborhood. The identified environmental determinants of a higher recreational walking time performed in one’s neighborhood may reflect perceived neighborhoods of larger sizes for the participants living in these environments (which would lead to a larger share of their walking activity in their perceived neighborhood). While we admit a possible bias, we do not believe these relatively small variations in the average size of perceived neighborhoods to be sufficient to drastically influence the reported time of recreational walking made in one’s residential neighborhood. What matters instead is whether there is or not a park around the residence that substantially increases recreational walking nearby the residence.

Finally, in addition to selection processes in the recruitment of the RECORD Cohort (convenience sample) [38], low educated and low income participants had higher odds to be excluded due to missing data in walking times (Additional file 1A). Adjustment of regression models on walking for education and income likely reduced selection biases. However, we could not examine whether participants and non-participants in RECORD, and whether participants excluded or not due to missing values in walking times differed in their true walking behavior.

### Relationships between the environment and recreational walking

For the two outcomes investigated, two independent associations were reported with neighborhood socioeconomic factors (with neighborhood education), three with the physical environment (one of them with green/open spaces, two with air traffic), and one with the service environment (density of destinations) (see the classification of environmental factors reported in Table 1).

Previous literature did not systematically observe associations between the socioeconomic environment and recreational walking. For example, one study documented an association between residing in a high socioeconomic status neighborhood and the practice of recreational walking even after adjustment for perceived environmental factors [60]. However, another study found that area socioeconomic status did not predict walking for recreation after adjustment for other environmental variables [61]. In our study, neighborhood socioeconomic effects were captured by education rather than income, in line with most previous research in RECORD on clinical risk factors of cardiovascular diseases [37,39,40,62,63]. A potential explanation is that a high average education of local residents may foster a general climate of values favorable to healthy lifestyles.

In relation to the physical environment, the presence and high quality of green/open spaces were associated with a higher recreational walking time in one’s residential neighborhood. Additional file 1B demonstrates that the relationship between the presence/quality of green/open spaces and recreational walking in one’s residential neighborhood was not attributable to same-source bias (i.e., to the fact that the ecometric variable was based on the aggregation of information provided by the participants who themselves reported their walking time). This finding is coherent with a recent review [11] that indicated that a positive relationship between parks/open spaces and recreational walking was reported in 44% of the cases [61,64,65]. The fact that an association was documented with the presence/quality of green/open spaces (shared perception, as aggregated through the ecometric approach) rather than with the objective surface of green spaces suggests that the quality of green/open spaces is important in addition to their availability, a conclusion on the importance of the quality of recreational facilities that also emerged from previous literature [11].

As also related to the physical environment, living in the air traffic area around the two international Paris Region airports (as objectively assessed) was inversely associated with recreational walking in two models. No previous study investigated the association between air traffic exposure and walking. A plausible hypothesis is that noise from air traffic around international airports discourages recreational walking, a situation of environmental injustice. While exposure to air traffic was associated with lower odds of recreational walking, other environmental nuisances such as the presence of a highway, road traffic pollution, or waste treatment facilities nearby the residence were not.

A recent review of previous literature identified recreational walking to be less dependent on destinations than utilitarian walking [11]. Apart from the density of destinations, previous studies have reported exercise/recreational walking to increase with housing [66], residential [8], and population [20] density and with composite walkability indices [67]. In our French context, in relation to the service environment, a high density of destinations and services nearby the residence was associated with a higher recreational walking time in one’s neighborhood (effects of density on recreational walking were better captured by service density than by densities of population, buildings, intersections, and public transportation stations). A potential interpretation is that there is no perfect separation between recreational and utilitarian walking and that hybrid walking episodes exist.

Although weather condition was associated with recreational walking, none of the weather variables interacted with the identified environmental effects. It suggests that these characteristics of the socioeconomic, physical, and service environment are supportive of recreational walking regardless of the weather.

It is, however, important to keep in mind that a large number of environmental factors that were tested were not associated with recreational walking in the final models. Overall, of the 50 associations with environmental factors that were tested (25 factors × 2 outcomes), 33 associations were documented when each environmental factor was introduced separately in the model without adjustment for other environmental factors (but with adjustment for individual and weather variables); but only 6 associations were documented when environmental predictors were mutually adjusted for. In addition to the factors not associated with walking discussed above, it should be noted that no association was documented with the social-interactional environment or with the symbolic environment. All the environmental factors that were associated were either already identified in previous literature (neighborhood socioeconomic status, density of destinations, green/open spaces); or identified in the different models, thus relatively consistent (neighborhood socioeconomic status, air traffic exposure); or related to plausible hypotheses (all factors, including air traffic exposure).

The associations between environmental factors and walking were of modest magnitude and had relatively wide confidence intervals. In the final models, none of the associations identified showed an odds ratio above 1.5 (or below 0.67). It should be noted, however, that the overall influence of the environments identified in the initial models was shared between the different environmental factors when they were considered simultaneously (i.e., the strength of the relationship of each environmental factor with walking was reduced when the different factors were adjusted for each other). An indicator of the overall disparities that were predicted by the different environmental factors suggests that the odds of reporting recreational walking and the odds of a higher neighborhood recreational walking time were, respectively, 1.59 times and 1.81 times higher in the most vs. the least supportive environments (lowest and highest quartiles of cumulated effects of the different environmental factors). Such an overall quantification of environmental influences on walking may be useful to convince policymakers that, in addition to information and health promotion campaigns, interventions that address the environmental determinants of walking are relevant.

More environmental predictors and stronger associations were documented for the recreational walking time in the neighborhood than for overall recreational walking, which was expected because in the first case both the exposure and the outcome refer to the residential neighborhood. Finally, it was found that environmental factors explained to a greater extent spatially structured than spatially unstructured variations between neighborhoods. This finding empirically confirms the usefulness of spatial-multilevel regression models that are able to isolate, in the unexplained between-neighborhood variation, a part of it that is spatially structured, which is useful to generate hypotheses. Such spatially structured neighborhood variations in walking were not completely eliminated by the environmental factors retained in the models. Our future work will have to build on the map of residual spatial variations in walking reported in the bottom part of Figure 2 to generate additional explanatory environmental hypotheses.

## Conclusions

Despite differences between utilitarian and recreational walking, our study suggests that improving the access to destinations and services in the neighborhood, through the interpenetration of residential and commercial uses of land, may be also beneficial for recreational walking.

This study also emphasizes the importance of providing green and open spaces of quality to promote recreational walking. While reducing the distance to and increasing the surface of green/open spaces imply costly efforts, less costly interventions implementable in the short term that improve the quality and the attractiveness of existing green/open spaces may also be effective in promoting recreational walking.

Finally, exposure to air traffic appeared as a previously unrecognized barrier to recreational walking. Our study therefore provides additional arguments that point to the need to mitigate the environmental nuisances associated with air traffic.

## Competing interests

The authors declare that they have no competing interests.

## Authors’ contributions

BC designed the overall study, conceived the design of the present analysis, analyzed the data, and wrote the paper. FT and BP contributed to the design of the overall study. CS, HC, JMO, and CW provided suggestions to orientate the analysis of the data. All co-authors critically revised the manuscript for important intellectual content. All authors read and approved the final manuscript.

## Supplementary Material

Additional file 1 **The environmental correlates of overall and neighborhood based recreational walking (a cross-sectional analysis of the RECORD Study). A** - Missing values in the walking variables and determinants of missingness. **B** - Description of the ecometric approach followed to measure some of the neighborhood variables. **C** - Cutoff values for the categorization of environmental variables in 4 classes comprising a similar number of participants. Click here for file
