# Supplementary material for: The environmental correlates of overall and neighborhood based recreational walking (a cross-sectional analysis of the RECORD Study)
Source: Int J Behav Nutr Phys Act. 2014 Feb 21;11:20. doi: 10.1186/1479-5868-11-20 (PMC3943269; doi:10.1186/1479-5868-11-20)
Supplement: Additional file 1 — The environmental correlates of overall and neighborhood based recreational walking (a cross-sectional analysis of the RECORD Study). A - Missing values in the walking variables and determinants of missingness. B - Description of the ecometric approach followed to measure some of the neighborhood variables. C - Cutoff values for the categorization of environmental variables in 4 classes comprising a similar number of participants. [file 1479-5868-11-20-S1.doc]

**Additional file 1**

**The environmental correlates of overall and neighborhood based recreational walking (a cross-sectional analysis of the RECORD Study)**

**Table of content**

[A – Missing values in the walking variables and determinants of missingness 2](#__RefHeading___Toc374995929)

[B – Description of the ecometric approach followed to measure some of the neighborhood variables 6](#__RefHeading___Toc374995930)

[C – Cutoff values for the categorization of environmental variables in 4 classes comprising a similar number of participants 9](#__RefHeading___Toc374995931)

[References 10](#__RefHeading___Toc374995932)

# A – Missing values in the walking variables and determinants of missingness

*Patterns of missingness of walking times*

In the RECORD Study questionnaire, 8 questions were asked on the walking behavior. As a preliminary step, patterns of missing values in these variables were investigated. Collecting approximate walking times in hours and minutes for 8 different destinations or purposes and locations was a very demanding task for the study participants and for the survey technicians.

The survey technicians and participants were instructed to indicate “0 h 00 mn” for each specific walking variable for which there was no walking time over the previous week. However, in the first months of the data collection process, a natural trend was observed in the participants to omit to indicate “0 h 00 mn” for one or several of the walking variables if they had not walked towards that specific destination or at that specific location.

It was therefore decided to make particular efforts to systematically check that the 8 walking time variables were correctly filled. Due to this reinforced procedure, a significant and regular decrease was observed in the average number of missing values for the 8 walking variables over the course of the study (see Figure S1, Jonckheere-Tepstra test: –14.1; p-value < 0.001)

**Figure S1 Average number of missing values by study year and month.**


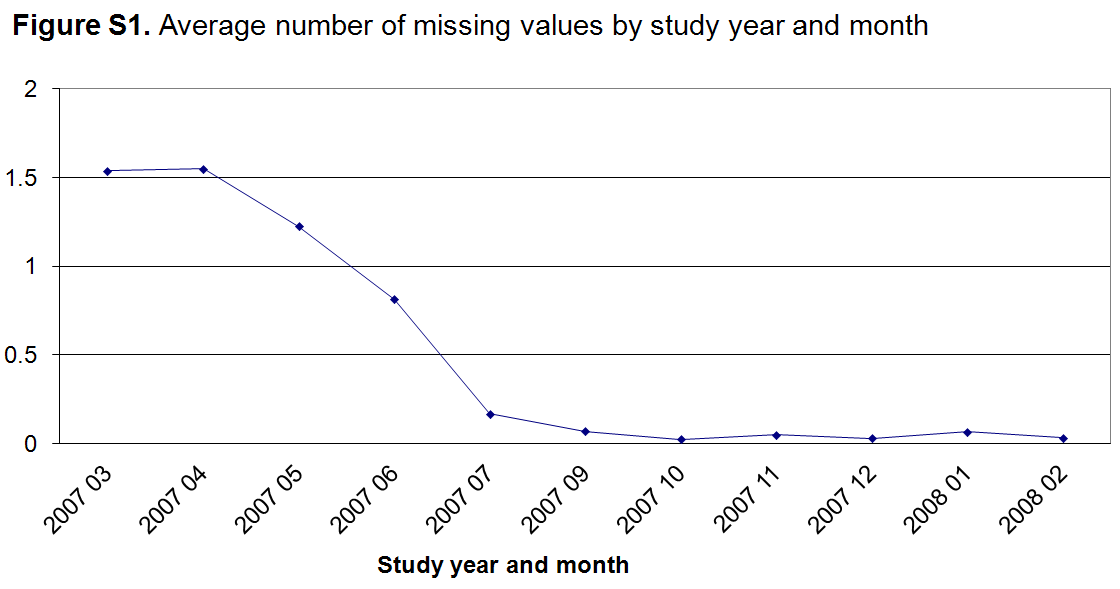


This change in the proportion of missing values over time is also obvious in Figure S2 representing the proportion of questionnaires with at least 1 missing value or with at least 4 missing values.

There is a fundamental ambiguity in the interpretation of missing values because it is not known whether a missing value corresponds to a walking time > 0 mn that was not reported or to the absence of walking time in this category over the previous 7 days.

Based on the experience of the researchers and survey technicians, it is clear that in most cases, missing values corresponded to “0 h 00 mn” of walking time, especially when the participants had provided walking time information for the other walking variables.

Under the assumption that all missing values corresponded to 0 mn of walking time in the specific category, no decrease would be observed over the course of the study in the average number of walking variables per questionnaire that are either equal to 0 or missing. However, Figure S3 suggests that the average sum of missing values and answers equal to “0 mn” per questionnaire decreased over time (Jonckheere-Tepstra trend test: –2.72; p-value < 0.05).

**Figure S2 Proportion of questionnaires with at least one or four missing values.**


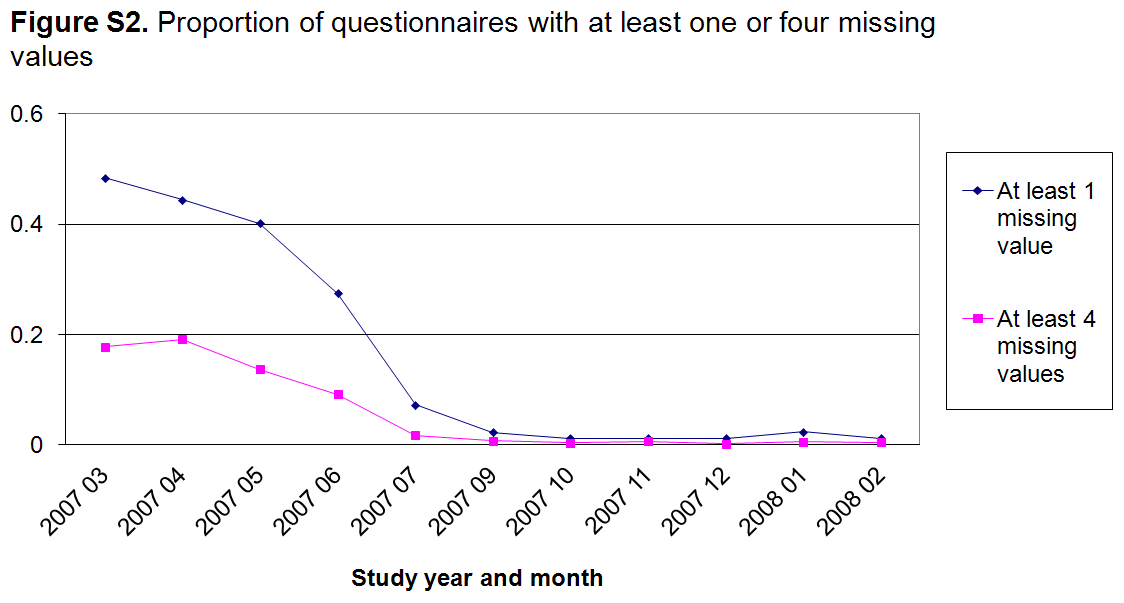


**Figure S3 Average number of walking time items per questionnaire with either a “0 mn” answer or a missing value in the overall sample.**


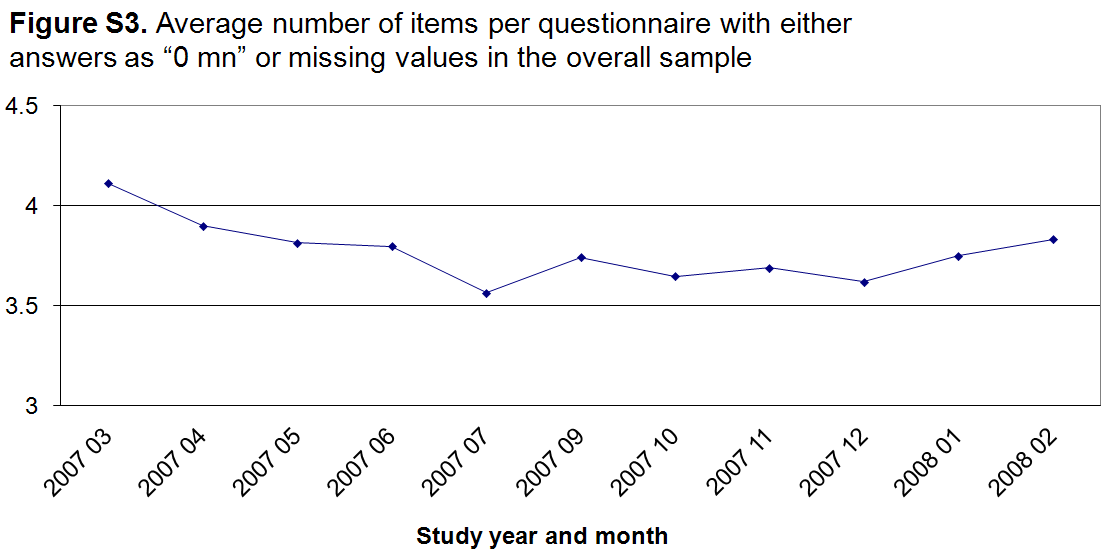


Therefore, the hypothesis that all the excess of missing values at the beginning of the study was attributable to non-reported “0 h 00 mn” of walking time may be inadequate. A perhaps insufficient verification of questionnaires at the beginning of the study led to an under-reporting of effective walking time.

*Exclusion of participants with an excessive number of missing values*

It was found reasonable to assume that, in questionnaires with only few (e.g., 1–3) missing values, missing values correspond to non-reported “0 mn” of walk, especially when the other reported values correspond to effective times of walk.

On the opposite, in the few questionnaires with a lot of missing values, it was assumed that there are higher odds that missing values correspond to an underreporting of effective walking times.

As a correction, it was therefore decided to remove from the analyses all the questionnaires that had an excessive number of missing values. As a sensitivity analysis, all questionnaires with at least 4 missing values (n = 274 questionnaires), all questionnaires with at least 5 missing values (n = 185 questionnaires), and all questionnaires with at least 6 missing values (n = 111 questionnaires) for the 8 walking time variables were successively excluded.

To evaluate the impact of these alternative scenarios of correction, a verification was to re-examine how the average sum of missing values and answers equal to “0 mn” per questionnaire changed over time after removing the 111 questionnaires, 185 questionnaires, or 274 questionnaires (see Figure S4). As shown in the Figure, this average number was still decreasing over the course of the study when removing the 111 questionnaires with at least 6 missing values (Jonckheere-Tepstra trend test: –1.77; p-value = 0.038). It suggests that probably too few incomplete questionnaires are removed from the dataset in this first scenario.

**Figure S4 Average number of walking time items per questionnaire with either a “0 mn” answer or a missing value, after excluding questionnaires with at least 4, 5, or 6 missing values.**


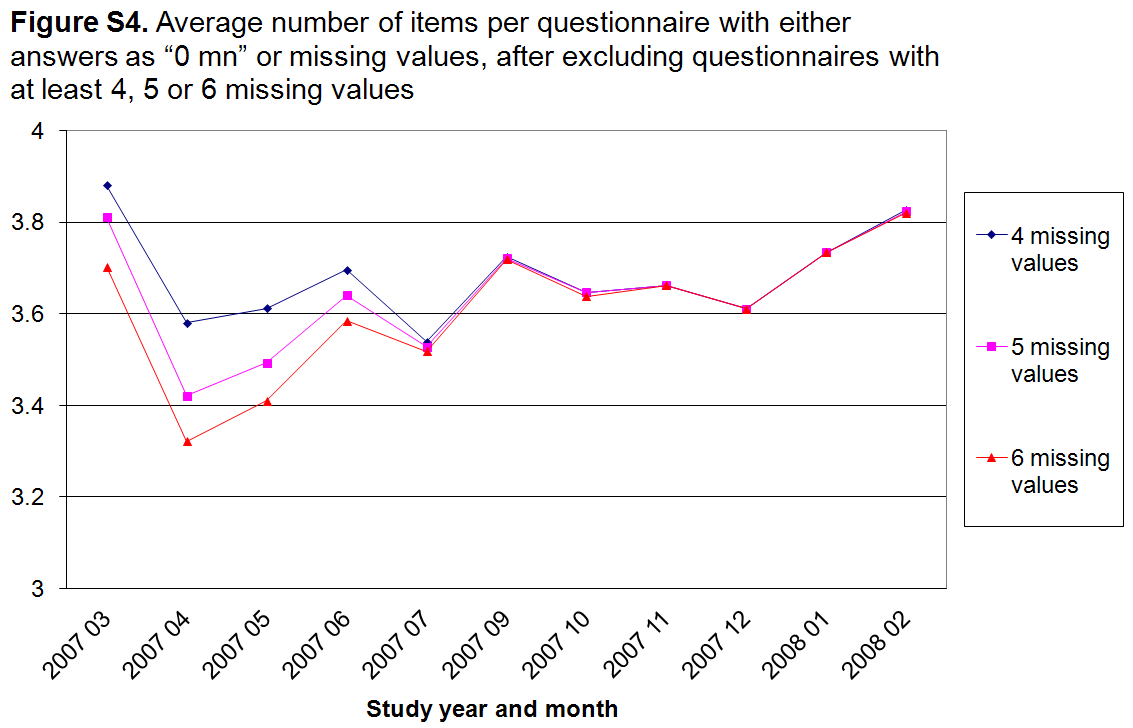


When removing questionnaires with at least 5 missing values, the average sum of items per questionnaire with a “0 mn” answer or a missing value no longer decreased over the course of the study as it did before removing the 185 questionnaires (the Jonckheere-Tepstra trend test lead to a statistic of –1.23 and to an associated p-value of 0.11, not allowing to conclude to a change in the average over the course of the study).

When removing the 274 questionnaires with at least 4 missing values, the Jonckheere-Tepstra trend test provided a statistic of –0.82 with an associated p-value of 0.21. Therefore, in this scenario as well, a decreasing trend could not be demonstrated over the course of the study in the average sum of items with a “0 mn” answer or a missing value.

In the end, it was decided to delete all questionnaires with at least 5 missing values (n = 185). After this correction, it was found reasonable to make the assumption that all missing values corresponded to non-reported “0 mn” of walking time. In the 7105 questionnaires retained for the analyses, all missing values were set to “0 h 00 mn” of walking time.

*Determinants of missingness of walking times*

We estimated a multilevel logistic regression model for being excluded from the analyses on walking behavior due to an excessive number of missing values in the walking times (binary outcome defined in the whole RECORD database and equal to 1 for 185 of the 7290 participants, otherwise equal to 0). The random effect was defined at the municipality level (121 broad geographic units including 10 districts of Paris and 111 other municipalities of the Ile-de-France Region). All the individual demographic and socioeconomic variables considered in the analyses for walking and selected neighborhood socioeconomic variables (neighborhood education and income) were considered as potential explanatory variables. Only the variables that remained associated with the outcome after mutual adjustment were retained in the final model. Age and sex were forced into the model.

As shown in Table S1 below, only individual education and household income were retained in the final model. The odds of an excessive number of missing values for walking times were much higher among low educated participants and higher among participants from low income households (after mutual adjustment).

| **Table S1 Associations between individual socioeconomic characteristics and the odds of exclusion from the analyses due to an excessive number of missing values in the walking times** | |
| --- | --- |
|  | **OR (95% CrI)** |
| **Age (vs. 30–44)** |  |
| 45–59 | 1.00 (0.73, 1.38) |
| 60–79 | 1.15 (0.79, 1.19) |
| **Male (vs. female)** | 0.94 (0.71, 1.26) |
| **Individual education (vs. upper tertiary)** |  |
| Higher secondary and lower tertiary | 1.18 (0.77, 1.82) |
| Primary and lower secondary | 1.93 (1.28, 2.89) |
| No education | 2.78 (1.73, 4.46) |
| **Household income (vs. high)** |  |
| Medium high | 0.91 (0.53, 1.56) |
| Medium low | 1.51 (0.94, 2.41) |
| Low | 2.25 (1.42, 3.56) |
| Legend: CrI, credible interval; OR, odds ratio. | |

# B – Description of the ecometric approach followed to measure some of the neighborhood variables

*Psychometric description of individual scales*

Different environmental dimensions investigated in the study were assessed with ecometric variables, each captured with different survey questions or items that reflect complementary aspects of the construct. As information on the psychometric reliability of the individual scales that were used to construct the ecometric environmental variables, we report in Table S2 the Cronbach alpha coefficient.

| **Table S2 Cronbach alpha coefficient for the individual scales related to the neighborhood ecometric variables** | |
| --- | --- |
| **Dimension** | **Cronbach alpha** |
| Presence and quality of green and open spaces (3 items) | 0.81 |
| Deterioration of the physical environment (4 items) | 0.82 |
| Neighborhood social cohesion (4 items) | 0.69 |
| Neighborhood stressful social interactions (5 items) | 0.75 |
| Neighborhood mistrust and hostility (5 items) | 0.68 |
| Stigmatized neighborhood identity (3 items) | 0.63 |
|  | |

*Description of the ecometric approach*

As indicated in Table 1 of the main article, a number of variables related to the physical environment, social-interactional environment, and symbolic environment were determined through the ecometric modeling strategy . In almost all cases, three-level (survey questions, individuals, TRIRIS areas) multilevel ordinal logit models were estimated with the RECORD participants’ answers to different questions about their neighborhood as the outcomes. Most ecometric variables were therefore grounded on different survey items that were intended to capture in a complementary way each of the environmental dimensions of interest. The only exception was the variable on the existence of a shared feeling of insecurity in the neighborhood, which was only based on a single survey item and therefore determined from a two-level (individuals, TRIRIS areas) multilevel model. Except perhaps for neighborhood social cohesion, there were a high intra-individual correlation and a high intra-TRIRIS correlation in the answers to the questions for each environmental dimension, suggesting that the scales were psychometrically and ecometrically sound . Multilevel models were employed to aggregate at the individual level the information provided by each respondent and to combine the answers of the different individuals of each neighborhood to construct indicators at the neighborhood level. As recommended, the TRIRIS-level random effect of each multilevel model was then used as an explanatory variable of walking behavior quantifying each dimension of the physical/social environment. In order to derive reliable environmental variables with this approach, it is necessary to have enough individuals per neighborhood assessing their environment. This is the reason why the ecometric measurement protocol was *a priori* conceived to be implemented, not at the most local neighborhood level (equivalent of census block groups), but at the level of slightly larger neighborhoods (TRIRIS areas, equivalent of census tracts). The distribution of the number of participants per TRIRIS neighborhood was as follows: median = 10; interquartile range = 6–14.

*Information on the reliability of the ecometric variables*

As additional information on the quality of the estimated neighborhood ecometric variables, we report both the intra-neighborhood correlation coefficient of the ecometric items and the reliability of each of the neighborhood measures (Table S3). The items could take 4 values from 1 to 4, expressing the extent to which the participant agreed with the sentence describing her/his own neighborhood. Multilevel ordinal regression models were estimated. The intra-neighborhood correlation coefficient was computed as the ratio of the neighborhood-level variance to the sum of the neighborhood-level and individual-level variances. As explained elsewhere , the neighborhood-level reliability of the neighborhood score is a function of the intra-neighborhood correlation and of the number of participants per neighborhood. Neighborhood reliabilities range from 0 to 1. The reliability is high when the intra-neighborhood correlation is strong and when the sample size per neighborhood is large.

| **Table S3 Intra-neighborhood correlation of individual answers and reliability of the neighborhood ecometric measures** | | |
| --- | --- | --- |
| **Ecometric scale** | **Intra-neighborhood correlation (95% CI)** | **Reliability** |
| Presence and quality of green and open spaces | 0.34 (0.31, 0.37) | 0.84 |
| Deterioration of the physical environment | 0.28 (0.25, 0.31) | 0.80 |
| Neighborhood social cohesion | 0.06 (0.04, 0.09) | 0.39 |
| Neighborhood stressful social interactions | 0.28 (0.25, 0.32) | 0.80 |
| Neighborhood mistrust and hostility | 0.17 (0.14, 0.20) | 0.67 |
| Stigmatized neighborhood identity | 0.30 (0.26, 0.34) | 0.81 |
| Legend: CI, confidence interval | | |

*Are the estimated associations between ecometric variables and walking attributable to same-source bias?*

A concern is that the associations documented between ecometric variables and walking may be attributable to same-source bias, i.e., would be driven by the fact that the value of the ecometric variable for an individual is shaped to some extent by the participant’s answers to the ecometric survey questions. In other words, the associations with ecometric variables would not reflect genuinely contextual effect but relationships between individual perceptions and walking.

An approach to investigate whether same-source bias is a concern is to remove from the database the information for a given individual when defining the value of the neighborhood ecometric variable for that participant. In practice, it means that for our sample of 7105 participants, it is necessary to re-estimate the ecometric multilevel model 7105 times, each time without one of the participants.

Estimating such an ecometric model with Markov chain Monte Carlo techniques in Winbugs takes more than 10 hours. It is therefore totally unrealistic to use this approach to estimate the 7105 models. Estimating 3-level models (items, individuals, neighborhoods) with the SAS Proc Mixed would be as well much too long (several hours for each model). The MLwiN software is much faster for estimating 3-level models but it does not easily allow one to automatize the process (more than 7000 models have to be estimated).

A practical option was to compute a score at the individual level as the sum of the ecometric items and estimate a two-level multilevel model with SAS Proc Mixed. An automated procedure in SAS allowed us to estimate 7105 times the multilevel ecometric model, each time with a sample that excludes one participant. The ecometric variable for each participant is the corresponding neighborhood-level random effect based on the sample from which the participant was excluded.

We reestimated the model for the neighborhood recreational walking time, in which an ecometric variable had been selected. In Table S4 below, we only report the association between the ecometric variable and the outcome, estimated from a model adjusted for individual sociodemographic characteristics and for the other environmental variables listed in Table 5 of the main article.

| **Table S4 Association between the presence and quality of green and open spaces (ecometric variable) and the recreational walking time in one’s neighborhood corrected from same-source bias** | |
| --- | --- |
|  | **OR (95% CrI)** |
| *Presence and quality of green and open spaces (vs. low)* |  |
| Medium low | 0.97 (0.85, 1.09) |
| Medium high | 1.01 (0.89, 1.15) |
| High | 1.22 (1.05, 1.42) |
| Legend: CrI, credible interval; OR, odds ratio. | |

This analysis shows that the association between the presence/quality of green/open spaces and the recreational walking time in one’s residential neighborhood persisted when using the corrected ecometric variable. The association was therefore not attributable to same-source bias.

# C – Cutoff values for the categorization of environmental variables in 4 classes comprising a similar number of participants

Most of the environmental variables were divided into 4 categories comprising a similar number of participants (except the presence of water nearby the residence, the presence of a highway nearby the residence, residing in an air traffic area, the presence of a waste treatment facility nearby, and the presence of a shopping center that were coded with binary variables; and the presence of monuments nearby that was divided into 3 categories: 0, 1 or 2, >2). Table S5 provides the cutoffs that were used to categorize the variables into 4 categories. All environmental variables categorized in 4 classes are listed in Table S5, except the ecometric variables for which the neighborhood random effect scale is not intrinsically meaningful.

| **Table S5 Cutoff values for the categorization of environmental variables in 4 classes comprising a similar number of participants** | | | |
| --- | --- | --- | --- |
| **Neighborhood characteristic** | **First quartile** | **Median** | **Third quartile** |
| **Neighborhood sociodemographic environment** |  |  |  |
| Neighborhood median income (annual household income by consumption unit in €) | 21138 € | 26244 € | 31189 € |
| Neighborhood education (proportion with a University degree, > BAC + 2) | 29% | 44% | 53% |
| Neighborhood population density (inhabitants per km²) | 7599 per km² | 13537 per km² | 27453 per km² |
| **Neighborhood physical environment** |  |  |  |
| Proportion of the neighborhood covered with buildings | 17% | 25% | 35% |
| Proportion of the neighborhood covered with green spaces | 2% | 5% | 8% |
| Density of street intersections (per km²) | 138 per km² | 160 per km² | 183 per km² |
| Link node ratio | 1.30 | 1.35 | 1.40 |
| Road traffic-related pollution (nitrogen dioxide, annual concentration in µg/m^3^) | 30 µg/m^3^ | 38 µg/m^3^ | 46 µg/m^3^ |
| **Neighborhood service environment** |  |  |  |
| Density of destinations (number of destinations) | 124 | 334 | 1053 |
| Number of transportation lines | 7 | 10 | 14 |
| Proportion of incoming and outgoing traffic by public transportation rather than car | 0.43 | 0.52 | 0.67 |
|  | | | |

# References

1. Raudenbush SW, Sampson RJ: **Ecometrics: Toward a Science of Assessing Ecological Settings, With Application to the Systematic Social Observation of Neighborhoods.** *Sociol Methodol* 1999, **29:**1-41.

2. Mujahid MS, Diez Roux AV, Morenoff JD, Raghunathan T: **Assessing the measurement properties of neighborhood scales: from psychometrics to ecometrics.** *Am J Epidemiol* 2007, **165**(8)**:**858-867.

3. Chaix B, Lindstrom M, Rosvall M, Merlo J: **Neighbourhood social interactions and risk of acute myocardial infarction.** *J Epidemiol Community Health* 2008, **62**(1)**:**62-68.

4. Mujahid MS, Diez Roux AV, Borrell LN, Nieto FJ: **Cross-sectional and longitudinal associations of BMI with socioeconomic characteristics.** *Obes Res* 2005, **13**(8)**:**1412-1421.
